# The E3 Ubiquitin-Protein Ligase Cullin 3 Regulates HIV-1 Transcription

**DOI:** 10.3390/cells9092010

**Published:** 2020-09-01

**Authors:** Simon Langer, Xin Yin, Arturo Diaz, Alex J. Portillo, David E. Gordon, Umu H. Rogers, John M. Marlett, Nevan J. Krogan, John A. T. Young, Lars Pache, Sumit K. Chanda

**Affiliations:** 1 Immunity and Pathogenesis Program, Infectious and Inflammatory Disease Center, Sanford Burnham Prebys Medical Discovery Institute, La Jolla, CA 92037, USA; simon.langer@boehringer-ingelheim.com (S.L.); xyin@sbpdiscovery.org (X.Y.); aportillo@atarabio.com (A.J.P.); uhrogers@ucsd.edu (U.H.R.); 2 Boehringer Ingelheim Pharma GmbH & Co. KG, 55216 Ingelheim am Rhein, Germany; 3 Department of Biology, La Sierra University, Riverside, CA 92515, USA; adiaz@lasierra.edu; 4 The Nomis Center for Immunobiology and Microbial Pathogenesis, The Salk Institute for Biological Studies, La Jolla, CA 92037, USA; marlett@salk.edu; 5 Atara Biotherapeutics, Inc., Thousand Oaks, CA 91320, USA; 6 Department of Cellular & Molecular Pharmacology, University of California, San Francisco, CA 94143, USA; david.gordon@ucsf.edu (D.E.G.); nevan.krogan@ucsf.edu (N.J.K.); 7 Gladstone Institutes, San Francisco, CA 94158, USA; 8 Quantitative Biosciences Institute (QBI), San Francisco, CA 94158, USA; 9 UC San Diego School of Medicine, University of California, San Diego, La Jolla, CA 92093, USA; 10 Roche Pharma Research and Early Development, Roche Innovation Center Basel, 4070 Basel, Switzerland; john.young.jy3@roche.com

**Keywords:** HIV-1, Cullin 3, ubiquitin protein ligase, NF-κB signaling, viral gene transcription

## Abstract

The infectious life cycle of the human immunodeficiency virus type 1 (HIV-1) is characterized by an ongoing battle between a compendium of cellular proteins that either promote or oppose viral replication. On the one hand, HIV-1 utilizes dependency factors to support and sustain infection and complete the viral life cycle. On the other hand, both inducible and constitutively expressed host factors mediate efficient and functionally diverse antiviral processes that counteract an infection. To shed light into the complex interplay between HIV-1 and cellular proteins, we previously performed a targeted siRNA screen to identify and characterize novel regulators of viral replication and identified Cullin 3 (Cul3) as a previously undescribed factor that negatively regulates HIV-1 replication. Cul3 is a component of E3-ubiquitin ligase complexes that target substrates for ubiquitin-dependent proteasomal degradation. In the present study, we show that Cul3 is expressed in HIV-1 target cells, such as CD4+ T cells, monocytes, and macrophages and depletion of Cul3 using siRNA or CRISPR/Cas9 increases HIV-1 infection in immortalized cells and primary CD4+ T cells. Conversely, overexpression of Cul3 reduces HIV-1 infection in single replication cycle assays. Importantly, the antiviral effect of Cul3 was mapped to the transcriptional stage of the viral life cycle, an effect which is independent of its role in regulating the G1/S cell cycle transition. Using isogenic viruses that only differ in their promotor region, we find that the NF-κB/NFAT transcription factor binding sites in the LTR are essential for Cul3-dependent regulation of viral gene expression. Although Cul3 effectively suppresses viral gene expression, HIV-1 does not appear to antagonize the antiviral function of Cul3 by targeting it for degradation. Taken together, these results indicate that Cul3 is a negative regulator of HIV-1 transcription which governs productive viral replication in infected cells.

## 1. Introduction

The human immunodeficiency virus type 1 (HIV-1) is the causative agent of the acquired immunodeficiency syndrome AIDS and, over the course of the last century, has infected more than 70 million people worldwide. Current challenges in battling HIV/AIDS include the lack of an effective vaccine and the persistence of long-lived cellular reservoirs of latent virus that are not eradicated under antiretroviral therapies (ART). To undergo efficient replication in vivo, HIV-1, which only expresses a small set of viral proteins [1,2], has remarkably adapted to thrive in the hostile environment of the human host. Key adaptative processes include the counteraction or tolerance of antiviral proteins [3,4], but also engagement of many cellular proteins, so-called dependency factors, to successfully complete the viral life cycle [2,5,6,7,8].

Members of the cullin family serve as essential scaffolding proteins in the ubiquitin-proteasome system (UPS) and represent an important example of how HIV-1 manipulates host proteins to create a more amenable cellular environment. The UPS consists of three central components termed E1, E2, and E3. After the small ubiquitin protein is activated by a ubiquitin-activating enzyme, E1, and transferred to a ubiquitin-conjugating enzyme, E2, a ubiquitin ligase, E3, finally binds both, a given substrate and the E2 enzyme and enables the transfer of one or several ubiquitin molecules to a given substrate. Protein ubiquitination can lead to protein degradation but can also trigger signaling events or influence cellular trafficking [9,10]. Consequently, Cullin-RING E3 ligases are involved in various physiological and/or pathological processes such as cell cycle regulation, protein quality control, DNA replication, transcription and tumorigenesis [11,12]. In humans, the cullin family consists of seven family members (Cul1 to 3, Cul4a, Cul4b, Cul5, and Cul7) and the closely related p53-associated parkin-like cytoplasmic protein (PARC) [13]. Cullins are the central platforms of Cullin-RING E3 ligase (CRL) complexes. HIV-1 is known to use its accessory proteins Vpu, Vif and Vpr to hijack the function of several CRLs to counteract the human immune system and create a cellular environment which is beneficial for the virus [14]. HIV-1 Vpu was shown to co-opt CRL1 to ubiquitinate and degrade CD4, the primary receptor of HIV-1 [15,16]. Additionally, HIV-1 Vif interacts with CRL5 to degrade various members of the antiviral APOBEC family [17,18] and HIV-1 Vpr exploits CRL4A to direct the DNA helicase HLTF for degradation [19,20,21]. Furthermore, HIV-2 and closely related simian immunodeficiency viruses (SIVs) hijack CRL4 with their accessory protein Vpx to ubiquitinate and degrade the antiviral protein SAMHD1 [22,23,24].

Previously, a targeted siRNA screen performed in our lab identified yet another CRL family member, Cullin 3 (Cul3), as a regulator of early-stage HIV-1 replication [25]. Interestingly, in contrast to dependency factors CRL1, -4, and -5, which support viral replication, Cul3 negatively regulated HIV-1 replication. Cul3 was previously shown to target cyclins for ubiquitination and subsequent degradation, thereby regulating phase transition in the cell cycle, and the deletion of the *Cul3* gene in mice was shown to cause embryonic lethality [11,26,27]. In association with its substrate adaptor protein Kelch-like ECH-associated protein 1 (KEAP1), Cul3 was further shown to selectively inhibit the NF-κB pathway by catalyzing IκB kinase β (IKKβ) ubiquitination and degradation, thus preventing the activation of NF-κB signaling [28]. Besides acting as a central transcription factor to mount an inflammatory response, NF-κB binds to specific binding sites in the HIV-1 long terminal repeats (LTR), the promotor region of the viral genome, and thus regulates viral gene transcription, replication, and latency in HIV-1 target cells [29,30,31,32].

Here, we performed genetic knockdown and knockout experiments using siRNA or CRISPR/Cas9, respectively, and show that Cul3 impedes viral replication in primary CD4+ T cells. Overexpressing Cul3 conversely inhibits viral infection in a dose-dependent manner. We further show that viral mRNA expression, but not the generation of early and late reverse transcription products (RT), is affected by Cul3, and this effect is independent of Cul3-mediated cell cycle transition. Finally, we find that the NF-κB/NFAT transcription factor binding sites in the viral LTR promotor region are crucial for Cul3-dependent regulation of viral gene expression. In summary, our results provide additional insights into host-pathogen interactions that impact HIV-1 replication through demonstrating that the E3 ubiquitin-protein ligase Cul3 negatively regulates the activation of NF-κB and thereby impedes viral replication through regulation of proviral transcription.

## 2. Materials and Methods

### 2.1. Cell Culture and Isolation of Primary Cells

#### 2.1.1. Immortalized Cell Lines

Human embryonic kidney 293T cells (HEK293T, RRID: CVCL_0063) were authenticated and obtained from the American Type Culture Collection (ATCC) and maintained in Dulbecco’s Modified Eagle Medium (DMEM) supplemented with 10% fetal bovine serum (FBS, Life Technologies, Carlsbad, CA, USA; Cat.# 10437028), 2 mM glutamine, 100 Units/mL of streptomycin and 100 Units/mL penicillin (Corning, Corning, NY, USA; Cat.# 30-002-CI or Thermo Fisher Scientific, Waltham, MA, USA; Cat.# 10378016). HEK293T cells were tested for mycoplasma contamination every two months. Only mycoplasma negative cells were used for this study.

#### 2.1.2. Primary Cells

Human peripheral blood mononuclear cells (PBMCs) were obtained by Ficoll density centrifugation from healthy anonymous blood donors. CD4+ T cells were negatively selected using magnetic beads as per manufacturer’s instructions (Human CD4+ T Cell Isolation Kit, Stemcell Technologies, Vancouver, BC, Canada; Cat.# 17952). CD4+ T cells were cultured in RPMI 1640 supplemented with 10% FBS, 100 I.U. Penicillin, 100 μg/mL Streptomycin, 2 mM L-glutamine, and 30 Units/mL of rIL-2 (Peprotech, Rocky Hill, NJ, USA; Cat.# 200-02). Cells were stimulated with Human T-Activator CD3/CD28 Beads (Thermo Fisher Scientific, Waltham, MA, USA; Cat.#11132D) following manufacturer’s instructions and expanded in the presence of 30 Units/mL of rIL-2. Cells were infected by spinoculation at 1200× *g* for 1.5 h in multi-well plates. Experiments with primary T-cells were repeated with cells from eight different donors. CD14+ monocytes were isolated using magnetic beads as per manufacturer’s instructions (EasySep Human Monocyte Isolation Kit, Stemcell Technologies, Vancouver, BC, Canada; Cat.# 19359). Primary macrophages were further differentiated from the purified monocytes with recombinant human GM-CSF (10 ng/mL).

### 2.2. Proviral Constructs and Production of Virus Stocks

Wild type and Vesicular Stomatitis Virus glycoprotein G (VSVg)-pseudotyped HIV-1 NL4-3 luciferase viral stocks were generated by transfection of HEK293T cells with 9 μg of proviral DNA and 4 μg of Vesicular Stomatitis Virus glycoprotein G (VSVg) DNA per 10 cm plate using 10 μg of polyethylenimine (PEI). The supernatant was removed 24 h post-transfection and replaced with fresh media (DMEM, 10% FBS, 2 mM glutamine, 100 Units/mL of streptomycin, 100 Units/mL penicillin, and 20 mM HEPES). Two days after transfection the supernatants were collected and treated with 1 μL of 1 M Magnesium Chloride and 1 μL rDNase (Macherey-Nagel, Düren, Germany; Cat.# 740963) per mL of supernatant collected for 1 h at room temperature. The supernatant was filtered (0.22 μM) and aliquoted in 50 mL conical tubes with a 5 mL TNE-25% sucrose cushion and centrifuged at 3000× *g* and 4 °C overnight. The following day, the supernatant was removed, leaving roughly 0.5 mL of volume in the tube containing the pelleted virus. The tubes were placed at 4 °C for 6 h. The concentrated virus was collected and aliquoted, snap-frozen using dry-ice/ethanol, and stored at −80 °C. The molecular clone pHIV-1 NL4-3 Env- luciferase lacks a functional envelope protein and encodes firefly luciferase instead of the accessory protein Nef (pHIV-1 NL4-3-Luc-E-, kindly provided by the NIH AIDS Research Program). VSV-G pseudotyped viral stocks of HIVΔUSF, HIVΔNFIL6, HIVΔNF-κB and HIVΔSTAT5 were obtained by transfecting HEK293T cells as described above.

### 2.3. Assays to Quantify Viral Infection

For quantitative chemiluminescent infection assays in HEK293T cells infected with the VSVg-pseudotyped HIV-1 NL4-3 luciferase reporter virus, 20,000 cells in 100 μL DMEM per well were plated in a 96-well plate and transfected with the appropriate siRNA (see Section 2.4 for details). 48 h post-transfection, cells were infected with VSVg-pseudotyped HIV-1 NL4-3 luciferase reporter virus for 24 h and three wells were assayed for firefly luciferase activity using the BrightGlo reagent (Promega, Madison, WI, USA) according to the manufacturer’s instructions. Three additional wells were assayed for cell number and cell viability using the CellTiter-Glo reagent (Promega, Madison, WI, USA). The results obtained were normalized for relative cell number. For CD4+ T cells, 40,000 cells in 100 μL RPMI were seeded per well in a 96-well plate and transfected with Accell siRNAs according to the manufacturer’s instructions (see Section 2.4 for details). 48 h post-transfection, cells were infected with VSVg-pseudotyped HIV-1 NL4-3 luciferase reporter virus by spinoculation at 1200× *g* for 60 min at 37 °C. Viral infection and cell viability were measured at 48 h post infection using BrightGlo and CellTiter-Glo as described above.

### 2.4. siRNA and cDNA Transfection

Hs_CUL3_5 (Target sequence: AACAACTTTCTTCAAACGCTA) and Hs_CUL3_9 (TCGAGATCAAGTTGTACGTTA) siRNAs as well as the non-targeting scrambled controls were purchased from Qiagen. HEK293T cells were reverse transfected with siRNA either targeting CUL3 or a non-targeting scrambled control using lipofectamin RNAiMAX transfection reagent (Invitrogen, Carlsbad, CA, USA; Cat.# 13778150) by reverse transfection with a final siRNA concentration of 12.5 nM. Primary CD4+ T cells were transfected with the Accell siRNAs (Thermo Fisher Scientific, Waltham, MA, USA) CUL3_17 siRNA (target sequence: CGGCAAACUCUAUUGGAUA) or non-targeting control #1 (UGGUUUACAUGUCGACUAA) according to manufacturer’s instructions for a final siRNA concentration of 100 nM.

### 2.5. Western Blotting

To check for protein expression by western blot, cells were harvested and washed once with ice-cold PBS prior to adding Pierce IP lysis buffer (Thermo Fisher Scientific, Waltham, MA, USA; Cat.# 87787) containing 0.2 mM PMSF and protease inhibitor cocktail (Millipore Sigma, Burlington, MA, USA; Cat.# P8340). Approximately 100 μL lysis buffer were added per 10^6^ cells. After reconstituting the cells in lysis buffer, samples were incubated on ice for 10 min with 5 s of vortexing every 2–3 min. Subsequently, samples were centrifuged at 20,000× *g* for 20 min at 4 °C. After centrifugation, total protein concentrations in the supernatant were determined using the Pierce BCA protein assay kit (Thermo Fisher Scientific, Waltham, MA, USA; Cat.# 23227) and adjusted to equal concentrations by adding the appropriate amount of lysis buffer. After adding sample buffer (NuPAGE, LDL sample buffer (4X), Thermo Fisher Scientific, Waltham, MA, USA; Cat.# NP0008) containing 5% 2-Mercaptoethanol (Millipore Sigma, Burlington, MA, USA; Cat.# M6250), samples were boiled at 95 °C for 5 min. Samples were separated on 4–12% Bis-Tris pre-cast gels (Thermo Fisher Scientific, Waltham, MA, USA; Cat.# WG1402BOX) and transferred to Immobilon-P PVDF membranes (Millipore Sigma, Burlington, MA, USA; Cat.# IPVH00010). Blocking was performed for 45 min at RT using Odyssey Blocking buffer (LI-COR, Lincoln, NE, USA; Cat.# 927-50000) and subsequent washing using 0.1% Tween-20 in Tris-buffered saline (TBS) (20 mM Tris base and 150 mM NaCl at pH 7.6). Primary antibodies against CUL3 (Cell Signaling Technology, Danvers, MA, USA: Cat.#2759, dilution 1:1000 in TBS + 5% milk; Thermo Fisher Scientific, Waltham, MA, USA; Cat.#PA5-17397 dilution 1:1000 in TBS + 5% milk), β-actin (Cat.# 4970, dilution 1:3000 in TBS + 5% milk), β-Tubulin (Cat.# 2128 dilution 1:3000 in TBS + 5% milk) and GAPDH (Cat.# 2118 dilution 1:5000 in TBS + 5% milk) were purchased from Cell Signaling Technologies (Danvers, MA, USA). Other primary antibodies used were against IFIT1 (Thermo Fisher Scientific, Waltham, MA, USA; Cat.# PA3-848, dilution 1:1000 in TBS + 5% milk), and HIV-1 p24 (Abcam, Cambridge, MA, USA; Cat.# ab9071, dilution 1:1000 in TBS + 5% milk). Labeled secondary antibodies were purchased from LI-COR (LI-COR Biosciences, Lincoln, NE, USA; dilution 1:20,000 in TBS + 5% milk). Immunoblots were analyzed using an Odyssey CLx imaging system with Image Studio 5 software (LI-COR Biosciences, Lincoln, NE, USA).

### 2.6. NF-κB and NFAT Luciferase Reporter Assay

To analyze the effects of Cul3 on NF-κB or NFAT signaling, HEK293T cells were seeded in a 96-well plate (15,000 cells per well in 100 μL DMEM) and, the following day, co-transfected in triplicates with 3, 10, 20 or 50 ng of pLX304_CUL3-V5, 20 ng of an NF-kB-responsive firefly luciferase reporter construct (described earlier [33]) or 50 ng of an NFAT-responsive firefly luciferase reporter construct (described earlier [34]) and 5 ng of a Renilla luciferase reporter plasmid using Lipofectamine 2000 (Invitrogen, Carlsbad, CA, USA; Cat.# 11668500) according to the manufacturer’s instructions. To induce NF-κB signaling, cells were treated 16 h post transfection with 2.7, 8.3 or 25 ng/mL of TNFα (Cell Signaling, Danvers, MA, USA; Cat.# 8902SC). 24 h post TNFα stimulation, firefly luciferase activity was determined and normalized to the activity of the Renilla luciferase control plasmid using the Dual-Glo^®^ Luciferase Assay System (Promega, Madison, WI, USA; Cat.# E2920). To induce NFAT signaling, cells were treated 24 h post transfection with 600 ng/mL Phorbol 12-myristate 13-acetate (PMA) (Sigma Aldrich, St. Louis, MO, USA; Cat.# P1585-1MG) and 1 mM of ionomycin (Sigma Aldrich, St. Louis, MO, USA; Cat.# 56092-82-1 I0634-1MG). 17 h post PMA and ionomycin stimulation, firefly luciferase activity was determined and normalized to the activity of the Renilla luciferase control plasmid using the Dual-Glo^®^ Luciferase Assay System (Promega, Madison, WI, USA; Cat.# E2920). The construct expressing LacZ-FLAG was used as control [35].

### 2.7. Mapping the Antiviral Effect of Cul3 to the HIV-1 Replication Cycle

Cells were seeded in triplicate wells at 2 × 10^4^/well in 96-well plates and transfected with the appropriate siRNA using the RNAiMax Reagent (Invitrogen, Carlsbad, CA, USA). 48 h post transfection, cells were infected with VSVg-NL4-3 Luc virus. To measure the amounts of reverse transcription intermediates in infected cells, DNA was harvested from infected cells 24 h post infection using qPCR lysis buffer (0.1 M Tris-HCl, pH8; 0.01 M EDTA; 0.002 M CaCl2; 0.01% Triton X-100; 0.01% SDS) with 1 mg/mL Proteinase K. HIV-1 Early RT primers: forward GTGCCCGTCTGTTGTGTGAC and reverse GGCGCCACTGCTAGAGATTT. HIV-1 Late RT primers: forward TGTGTGCCCGTCTGTTGTGT and reverse GAGTCCTGCGTCGAGAGATC. To measure HIV-1 mRNA levels, total RNA was isolated using the Cells-to-CT kit according to the manufacturer’s instructions, followed by DNase treatment (Life Technologies, Carlsbad, CA, USA). cDNA was generated using the Cells-to-CT kit according manufacturer’s instructions. HIV-1 Late RT primers: forward TGTGTGCCCGTCTGTTGTGT and reverse GAGTCCTGCGTCGAGAGATC. The expression of viral and host transcripts was measured by quantitative real time PCR (qRT-PCR) analysis using a ViiA 7 instrument (Life Technologies, Carlsbad, CA, USA) with standard cycling conditions (95 °C for 3 min and 40 cycles of 95 °C for 20 s, 60 °C for 30 s), using the Fast SYBR Green Master Mix (Life Technologies, Carlsbad, CA, USA) according to the manufacturer’s instructions with 1 µM of each primer. The number of molecules in each reaction was determined by comparison to standard curves generated from the amplification of plasmid DNA containing the target sequence and normalized to the housekeeping genes PBGD or GAPDH. GAPDH primers: Forward CATGAGAAGTATGACAACAGCCT and Reverse AGTCCTTCCACGATACCAAAGT. PBGD primers: Forward AAGGGATTCACTCAGGCTCTTTC and Reverse GGCATGTTCAAGCTCGTTGG.

### 2.8. CRISPR/Cas9 Gene Knock Out in Primary CD4+ T Cells

#### 2.8.1. Polyclonal Knockouts in Primary CD4+ T-Cells

Polyclonal knockouts were performed in primary CD4+ T-cells as previously described [36,37]. Blood from healthy donors was harvested from Trima Leukoreduction chambers (Blood Centers of the Pacific, San Francisco, CA, USA). Primary CD4+ T-cells were purified by positive selection using a Fabian automated purification system (IBA Lifesciences) and stimulated by plate-bound CD3 and suspension CD28 (5 µg/mL) for 48–72 h prior to nucleofection. To generate Cas9 ribonucleoprotein complexes (Cas9-RNPs), 1 µL crRNA and 1 µL tracrRNA (160 µM each) were mixed and incubated for 35 min at 37 °C to make 80 µM guide RNA, then 2 µL Cas9 nuclease (40 µM) was added with mixing motion to make 4 µL of Cas9-RNP complex and incubated at 37 °C for an additional 20 min. Cas9-RNP complexes were electroporated into 300,000 stimulated primary CD4+ T-cells using the Lonza Primary P3 nucleofection kit and a 96-well shuttle connected to a 4D Nucleofector system. After nucleofection, cells were re-stimulated using T-cell activation and expansion beads and 80 IU/mL IL-2 IS (Miltenyi Biotec, Bergisch Gladbach, Germany), and cells were split every 2–3 days. Virus infections were performed 6 days post electroporation. Polyclonal knockout efficiency was assayed by western blotting.

#### 2.8.2. Virus Production

HIV-1 NL4-3 Nef:IRES:GFP virus was produced using the commercial transfection reagent PolyJet (Signagen, Frederick, MD, USA). Virus plasmid (12 µg) and 100 µLpolyJet were diluted separately in two tubes of 625 µL serum-free DMEM, then dilute PolyJet was added to dilute DNA and vortexed to mix. After 15 min at RT the transfection complexes were added to T175 flasks containing HEK293T/17 cells, and were gently rocked to mix. Virus was harvested 48 h post transfection, spun at 400× *g* for 5 min and filtered through a 0.45 µm filter. For every flask of cells 25 mL viral supernatant was precipitated by addition of 2.4 mL 4M NaCl and 5.5 mL 50% PEG-6000 for 2 h at 4 °C. Precipitated virus was pelleted for 40 min at 3500 rpm, resuspended in complete RPMI media at 50-fold concentration, and frozen in aliquots.

#### 2.8.3. HIV-1 Infection Quantitation by Flow Cytometry

Primary CD4+ T-cells were infected with concentrated Nef:IRES:GFP reporter virus in 96-well U-bottom plates. Cells were fixed 72 h post infection by addition of 2–4% formaldehyde in PBS, and percent infection was quantified based on GFP expression. Flow cytometry was performed using an Attune NxT flow cytometer equipped with a plate sampler (Thermo Fisher Scientific, Waltham, MA, USA).

### 2.9. Quantification of Cul3 Expression by qRT-PCR

Cul3 mRNA levels in HEK293T cells were determined by qRT-PCR as described in Section 2.7 using the following primers: Cul3 Forward TCGTAGACAGAGGCGCAATAA and Reverse GGCAGTGCATCACTCGTTCT.

### 2.10. Flow Cytometry

To quantify Cul3 protein levels by flow cytometry in HEK293T cells that were transfected with proviral DNA, cells were harvested 42 h post transfection, washed once with ice-cold PBS followed by fixation and permeabilization using a fixation/permeabilization solution kit according to the manufacturer’s protocol (BD Biosciences, Franklin Lakes, NJ, USA; Cat.# 554714). Cells were then intracellularly stained with an unconjugated antibody against Cul3 (Cell Signaling Technologies, Danvers, MA, USA; Cat.# 2759) or rabbit IgG Isotype control (abcam, Cambridge, MA; Cat.# ab172730) at 1.35 μg/mL for 30 min at RT. Samples were then washed and stained with a FITC-conjugated antibody against HIV-1 core antigen (Beckman Coulter, Indianapolis, IN, USA; Cat.# 6604665) at 4 μg/mL and an anti-rabbit Alexa Fluor 647-labeled secondary antibody (Thermo Fisher Scientific, Waltham, MA, USA; Cat.# A-21244) at 2 μg/mL for 30 min at RT in the dark. Cells were washed and resuspended in 300 μL PBS containing 1% BSA, 0.1% NaN3, and 1 mM EDTA. Flow Cytometry was performed on an Attune NxT flow cytometer (Thermo Fisher Scientific, Waltham, MA, USA) and data was analyzed with FlowJo software (FlowJo, Ashland, OR, USA). For cell cycle analysis, HEK293T cells were transfected with the indicated siRNAs, collected three days post transfection and fixed in absolute ethanol at −20 °C overnight. Cells were washed twice with cold PBS and resuspended in 3.8 mM sodium citrate, 40 µg/mL propidium iodide, and 0.5 µg/mL RNase A in DPBS. After incubating at 4 °C for 3 h, flow cytometry was performed on a BD FACScan (BD Biosciences, San Jose, CA, USA).

### 2.11. Statistical Analyses

Statistical significance was determined using an unpaired Student’s t test, using the GraphPad Prism software (version 8.2.0). Unless otherwise noted, n.s = not significant; * *p* < 0.05; ** *p* < 0.01.

## 3. Results

### 3.1. Cul3 Restricts Viral Replication in Single Cycle Assays

**Figure 1:**
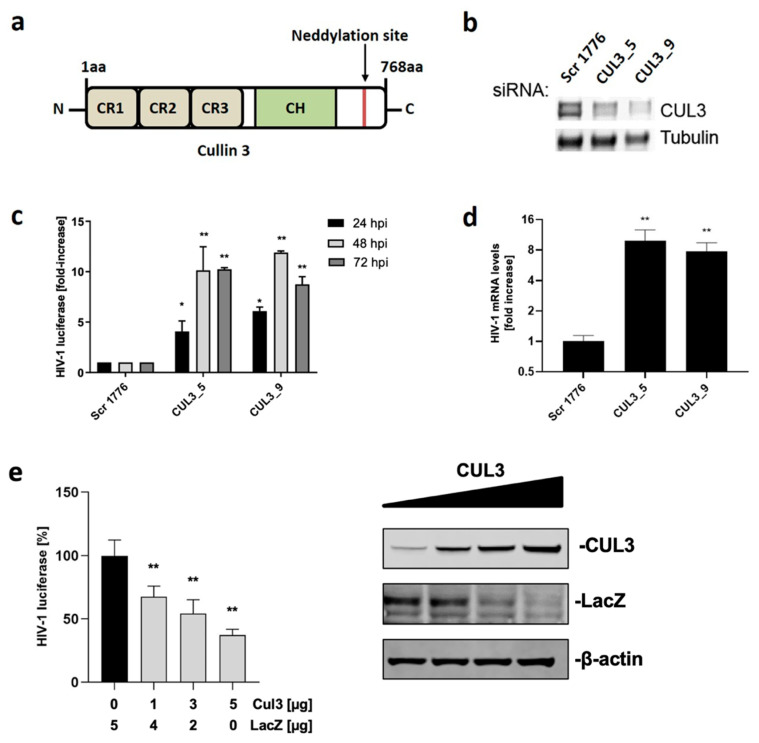
Cul3 impedes viral replication. (**a**) Cul3 domain organization with three cullin repeat domains (CR1-3) that enable interaction with a substrate receptor, a cullin homology domain (CH), which recruits an E2-enzyme and a neddylation site that regulates activity of the Cullin-RING E3 ligase complex. (**b**,**c**) HEK293T cells were reversely transfected with two independent siRNAs targeting Cul3 (CUL3_5 and CUL3_9) or a non-targeting siRNA (Scr 1776) as control. 48 h after transfection, cells were infected with a firefly luciferase-encoding HIV-1 NL4-3 (VSVg-pseudotyped). Cells were either harvested 24 h post infection to check for Cul3 protein expression by western blot (**b**), or harvested after 24, 48, or 72 h to measure viral gene expression by quantifying luciferase signal (**c**) (*n* = 2 +/− SD). (**d**) HEK293T.CD4.CCR5 cells were reverse-transfected with two independent siRNAs targeting Cul3 (CUL3_5 and CUL3_9) or a non-targeting siRNA (Scr 1776) as control. 48 h after transfection, cells were infected with wild type HIV-1 NL4-3. 48 h post infection, cells were harvested, viral mRNA was isolated, and expression of viral mRNA was quantified using qRT-PCR (*n* = 3 +/− SD). (**e**) HEK293T cells were co-transfected either with the indicated amounts Cul3 or a LacZ expressing vector as control and a Renilla luciferase control plasmid. 20 h post transfection, cells were infected with a firefly luciferase-encoding HIV-1 NL4-3 (VSVg). 48 h post infection, firefly luciferase activity was determined and normalized to the activity of the Renilla luciferase control plasmid (*n* = 3 +/− SD). * *p* < 0.05; ** *p* < 0.01.

Previously, we performed a targeted siRNA screen and identified Cul3 as a factor that impedes HIV-1 replication [25]. Except for Cul7 and PARC, all cullin proteins harbor three cullin repeat domains (CR1-3) which allow for interaction with substrate receptors that bind a specific substrate. Additionally, a cullin homology domain (CH), located near the C-terminus, is crucial for the interaction with a RING-finger protein that binds to a ubiquitin-charged E2 enzyme (Figure 1a). Activation of CRL is regulated by a post-translational modification at the C-terminus, called neddylation. If the ubiquitin-like protein NEDD8 (Neural precursor cell expressed developmentally down-regulated protein 8) is attached to the CRL, it is activated, whereas removing NEDD8 leads to inactivation. Building upon initial evidence that Cul3 impedes viral replication, we validated this effect using an siRNA approach. Cul3 is a 768 amino acid (Figure 1a) long, ~89 kDa, protein that is endogenously expressed in HEK293T cells. Endogenous expression levels of Cul3 in HEK293T cells were markedly reduced upon transfection with two independent siRNAs that target different regions of Cul3 (Figure 1b).

Compared to cells transfected with scrambled siRNA, Cul3 knock down increased HIV-1 luciferase expression up to 10-fold in cells that were infected for 24, 48 or 72 h with a VSVg-pseudotyped HIV-1 NL4-3 reporter virus that expresses a firefly luciferase (Figure 1c). Since a VSGg-pseudotyped virus takes an entirely different route of entry into cells compared to wild type HIV-1, we went on to explore the effect of Cul3 on HIV-1 infection in the context of the natural entry route. Therefore, we took advantage of an HEK293T cell line that has been modified to express the HIV-1 primary receptor CD4 as well as the coreceptor C-C chemokine receptor type 5 (CCR5) (HEK293T.CD4.CCR5). Analysis by qRT-PCR in the context of wild type HIV-1 infection revealed an 8-fold increase of viral mRNA expression after Cul3 knock down compared to cells treated with control siRNA (Figure 1d). This result is in line with the previous results using VSVg-pseudotyped virus. To validate the effect of Cul3 on HIV-1 infection using an orthogonal approach, we overexpressed increasing amounts of Cul3 cDNA in HEK293T cells and infected the cells using a VSVg-pseudotyped HIV-1 NL4-3 reporter virus. Transfecting HEK293T cells with increasing amounts of Cul3 cDNA resulted in elevated Cul3 protein levels (Figure 1e, right panel) and decreased HIV-1 infection in a dose-dependent manner up to 75% (Figure 1e, left panel). However, HIV-1 does not appears to antagonize the antiviral function of Cul3 by targeting it for degradation. As shown in Figure S1, endogenous Cul3 protein levels were not affected by the presence of HIV-1 NL4-3 or two primary HIV-1 isolates (STCO and CH293), which represent the most prevalent subtypes worldwide [38,39]. Translational shut-off experiments further showed that HIV-1 infection has no effect on the turnover levels of Cul3 proteins (Figure S1C), suggesting that HIV-1 infection does not mediate Cul3 degradation to antagonize its antiviral activity.

### 3.2. Cul3 Is Expressed in HIV-1 Target Cells and Is Not Type I IFN Inducible

**Figure 2:**
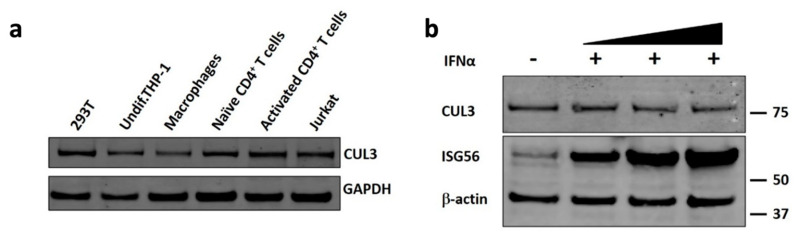
Cul3 is highly expressed in HIV-1 target cells and not induced by type 1 IFN. (**a**) Western blot analysis of Cul3 protein levels in HEK293T, Jurkat, THP-1, primary CD4+ T cells and primary macrophages. GAPDH served as loading control. (**b**) Western blot analysis of Cul3 protein levels in control and IFN-α-A/D-treated (1000 U/mL) THP-1 cells. ISG56 served as positive control and β-actin served as loading control.

HIV-1 displays a very distinct cell tropism and only infects haematopoietic cells that express the primary receptor CD4 and either one of the co-receptors CCR5 or CXCR4. These receptor combinations specifically occur in CD4+ T cells, macrophages and dendritic cells (DCs) [40]. To establish if Cul3 is expressed in the cell types that are relevant in the context of HIV-1 infection, we analyzed protein expression by western blot in an immortalized T cell line (Jurkat cells) and an immortalized monocytic cell line (THP-1 cells). Additionally, we analyzed Cul3 expression in primary CD4+ T cells and macrophages that were obtained from healthy blood donors. We also included HEK293T cells for comparison. As shown in Figure 2a, Cul3 is expressed in all of the investigated cell types, albeit to different degrees. Importantly, Cul3 was detected in activated CD4+ T cells, the primary target of the virus, which further supports a role of this factor in regulating HIV-1 infection.

Since the expression of many antiviral proteins is regulated by interferons, we also evaluated whether Cul3 expression is influenced by interferon. Treatment of THP-1 cells with IFNα drastically increased expression of the IFN-stimulated gene 56 (ISG56, also known as IFIT1), a protein well known to be upregulated upon interferon treatment. However, compared to ISG56, Cul3 protein levels remained unchanged upon IFNα treatment at all concentrations that were tested (Figure 2b).

### 3.3. Cul3 Restricts Viral Replication in Primary HIV-1 Target Cells

**Figure 3:**
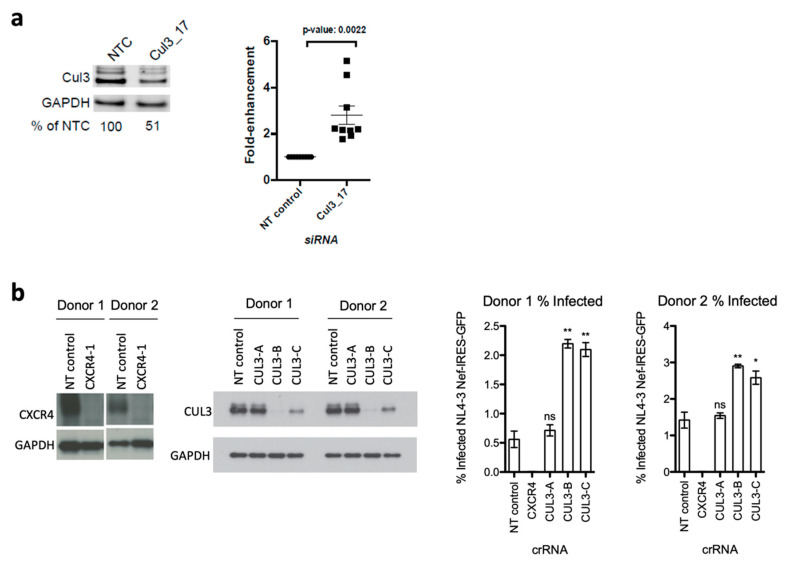
Cul3 siRNA knock down and CRISPR/Cas9 knockout in primary CD4+ T cells increase HIV-1 infection. (**a**) Primary CD4+ T cells were isolated from nine independent blood donors and transfected with an Accell Cul3-targeting siRNA (Cul3_17) or a non-targeting control siRNA (NT control). 48 h post transfection, cells were infected by spinoculation with a VSVg-HIV-1 NL4-3 luciferase reporter virus. 48 h post infection, luciferase activity and cell viability were quantified using BrightGlo and CellTiter-Glo, respectively. Data is plotted as fold-change compared to NT control (*n* = 9). A representative Western blot from one donor is shown. (**b**) Primary CD4+ T cells, isolated from two independent blood donors, were electroporated with Cas9/gRNA ribonucleoprotein complexes (Cas9-RNPs) that target either Cul3 (three different guide RNAs: CUL3-A-C), CXCR4 or a non-targeting control (NT control). After nucleofection, cells were expanded for additional six days and, during that time, split every two to three days. Six days post electroporation, cells were either harvested to analyze knockout efficiency by western blot or infected with concentrated HIV-1 NL4-3 GFP reporter virus. 72 h post infection, percent infection was quantified based on GFP expression using flow cytometry. Data is plotted as mean of three technical replicates +/− SD. * *p* < 0.05; ** *p* < 0.01.

After we verified that Cul3 is expressed in cells of the human immune system that are relevant for HIV-1 infection, we next sought to investigate the effect of Cul3 on HIV-1 replication in infected primary CD4+ T cells. Primary CD4+ T cells were isolated from healthy blood donors and transiently transfected with a non-targeting control siRNA (NT control) or a Cul3-targeting siRNA (Cul3_17). Subsequently, the cells were infected with an HIV-1 NL4-3 luciferase reporter virus. Luciferase expression was quantified 48 h post infection. Knock down efficiency was analyzed by western blotting and showed approximately 50% reduction of Cul3 protein expression (Figure 3a, right panel). On average, silencing of Cul3 increased HIV-1 luciferase expression by 3-fold compared to the non-targeting siRNA (Figure 3a, left panel). Interestingly, several specific bands were detected when probing the membrane with an antibody raised against Cul3. Consistent with previous reports, the additional bands with a higher molecular weight likely represent neddylated forms of Cul3 [41].

To further validate the antiviral effects of Cul3 in primary cells, we took an alternative approach, using a CRISPR/Cas9 knock out system in primary CD4+ T cells (Figure 3b). Therefore, we generated polyclonal knockouts by electroporation of Cas9-ribonucleoprotein (Cas9-RNP) complexes as previously described [36,37]. Specifically, we purified CD4+ T cells from healthy donors and electroporated Cas9-RNPs with three different guide RNAs targeting the Cul3 locus or the HIV-1 co-receptor CXCR4 as positive control or a non-targeting (NT) negative control. These cells were then either infected with an HIV-1 NL4-3 GFP reporter virus or lysed and analyzed by western blotting for Cul3 expression. Two of the three guide-RNAs targeting the Cul3 locus (CUL3-B and CUL3-C) generated a notable protein knockout. As expected, knockout of CXCR4 drastically decreased the number of HIV-1 infected cells compared to the NT control, whereas Cul3 knockout correlated with a robust increase of HIV-1 infected cells, as measured by flow cytometry 72 h post infection.

### 3.4. Cul3 Negatively Regulates HIV-1 Transcript Levels

After validating the Cul3 effect on HIV-1 in primary cells, we sought to identify the step in the viral life cycle Cul3 interferes with viral replication. To this end, HEK293T cells were transiently transfected with two independent siRNAs targeting Cul3 (CUL3_5 and CUL3_9), or a non-targeting (NT) control and subsequently infected with VSVg-pseudotyped HIV-1 NL4-3. Subsequently, expression of HIV-1 early reverse transcripts (defined as those generated prior to minus-strand DNA transfer, “Early RT”) and late transcripts (defined as those generated subsequent to minus-strand DNA transfer, “Late RT”) as well as viral mRNA were analyzed by qRT-PCR.

**Figure 4:**
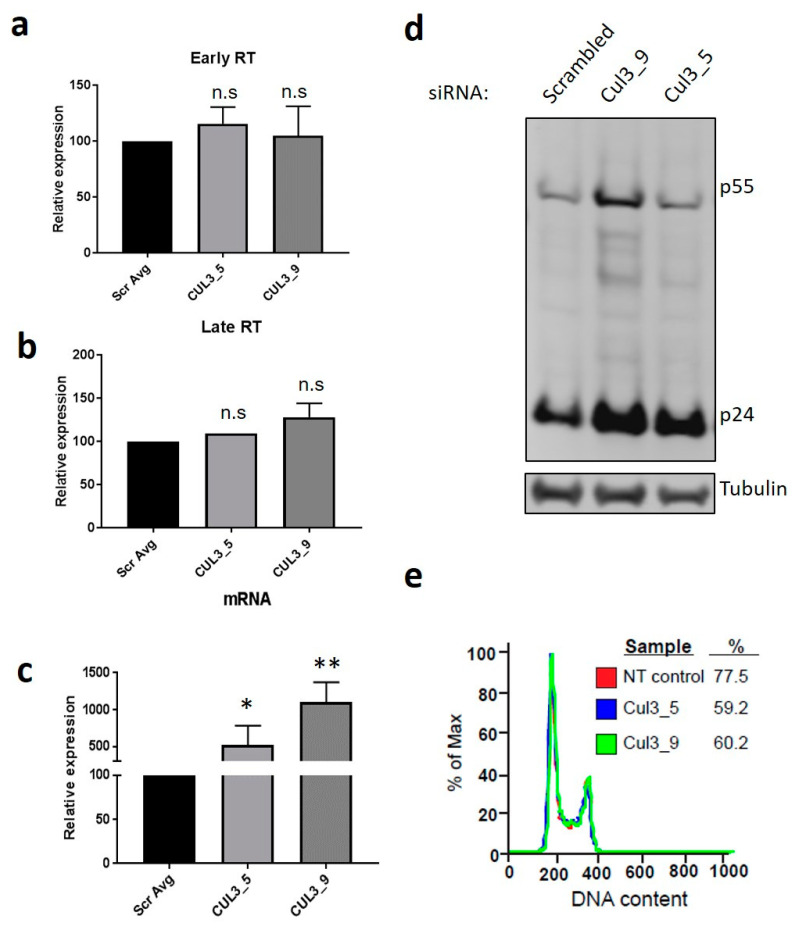
Cul3 reduces HIV-1 mRNA expression. (**a**,**b**) To measure the amounts of reverse transcription intermediates in infected cells, HEK293T cells were transfected with two independent siRNAs targeting Cul3 (CUL3_5 and CUL3_9) or a non-targeting siRNA as control (Scr). 48 h post transfection, cells were infected with a VSVg-pseudotyped HIV-1 NL4-3 luciferase reporter virus. 24 h post infection, cells were harvested and DNA was isolated and analyzed by qRT-PCR, using specific primers to amplify HIV-1 early or late RT (*n* = 1–3 +/− SD). (**c**) To analyze HIV-1 mRNA levels, total RNA was isolated 24 h post infection, reversely transcribed into cDNA and analyzed by qRT-PCR (*n* = 6 +/− SD). (**d**) To analyze virus particle release, HIV-1 p55 and p24 levels in the supernatant of HEK293T cultures were measured by western blot. (**e**) HEK293T cells were transfected with two independent siRNAs targeting Cul3 (Cul3_5 and Cul3_9) or a non-targeting (NT) siRNA as control. 48 h post transfection cells were harvested and cell cycle transition was analyzed by FACS using propidium iodine staining (*n* = 2). * *p* < 0.05; ** *p* < 0.01; n.s.: not significant.

Whereas Cul3 knock down did not alter the levels of early and late transcripts compared to the negative control (Figure 4a,b), silencing Cul3 significantly increased viral unspliced mRNA levels 5–10 fold and enhanced virus production in the supernatants, depending on the siRNA that was used (Figure 4c,d). This suggested the transcription of viral genes as the crucial step where Cul3 interferes with viral replication. Since Cul3 was shown to regulate the cell cycle transition from the G1 to the S phase by targeting cyclin E for ubiquitination and subsequent degradation [26], we investigated if the effect we observe on viral mRNA transcription is a consequence of this activity. A FACS-based cell cycle analysis in HEK293T cells, transfected with siRNAs targeting Cul3 (CUL3_5 and CUL3_9), or a non-targeting (NT) control, revealed no difference in DNA content in absence or presence of Cul3 (Figure 4e), indicating that the effect of Cul3 on HIV-1 gene expression is independent from its reported capability to regulate the G1/S cell cycle transmission.

### 3.5. NF-κB/NFAT Sites in the HIV-1 LTR Are Important for the Inhibition of HIV-1 Transcription by Cul3

**Figure 5:**
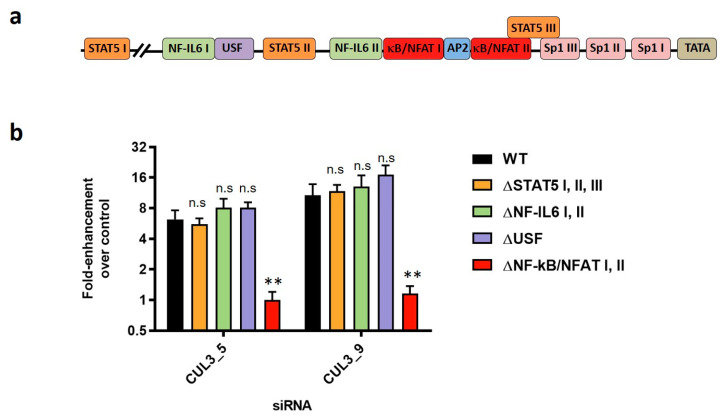
NF-κB/NFAT binding sites in the viral LTR are required for Cul3 to reduce HIV-1 mRNA expression. (**a**) Schematic representation of the HIV-1 LTR promotor region with various transcription factor binding sites. (**b**) HEK293T cells were transiently transfected with two independent siRNAs targeting Cul3 (CUL3_5 and CUL3_9) or a non-targeting siRNA as control. 48 h after transfection, cells were infected with a wild type VSVg-pseudotyped HIV-1 NL4-3 luciferase reporter virus (WT) or mutants thereof, in which the binding sites of various transcription factors within the viral LTR have been abrogated (∆STAT5 I, II, III; ∆NF-IL6 I, II; ∆USF and ∆NF-κB/NFAT I, II). 24 h post infection, viral mRNA expression was analyzed by qRT-PCR. Data is plotted as fold-enhancement compared to non-targeting control siRNA (*n* = 2–3 +/− SD). ** *p* < 0.01; n.s.: not significant.

The viral LTR represents the promotor region of the HIV-1 genome that drives viral gene expression. LTR-driven transcription of viral genes is mainly promoted by the viral regulatory protein transactivator of transcription (Tat) [42,43]. Additionally, the LTR harbors multiple binding sites for cellular transcription factors (Figure 5a) and viral gene expression, but also HIV-1 reactivation from the viral reservoir in latently infected primary memory CD4+ T cells, was shown to be partly dependent on the activity of these cis-acting elements [44,45,46,47,48,49,50]. One of the transcription factors that HIV-1 depends on to undergo efficient replication is the inflammatory transcription factor NF-κB [30].

We further find that overexpressing Cul3 in HEK293T cells reduces NF-κB activation in a dose-dependent manner (Figure S2). Based on these findings, we speculated that Cul3 might exert its effect on viral gene expression through modulation of NF-κB activation. To test this hypothesis, we took advantage of HIV-1 NL4-3 proviral constructs where the binding sites of transcription factors in the U3 region of the 5′ LTR have been abrogated [44]. Specifically, the three STAT5 binding sites (∆STAT5 I, II, III), the two NF-IL6 sites (∆NF-IL6 I, II), the upstream stimulating factor (∆USF) binding site or the two NF-κB/NFAT sites (∆NF-κB/NFAT I, II) in the viral LTR were abrogated. In line with previous results, depletion of Cul3 in HEK293T cells increased infection with wild type HIV-1 NL4-3 but also with HIV-1 ∆STAT5 I, II, III, ∆NF-IL6 I, II and ∆USF up to 15-fold. Interestingly, depletion of Cul3 in HEK293T cells did not alter infection with the HIV-1 ∆NF-κB/NFAT I, II mutant, suggesting that the NF-κB/NFAT binding sites in the LTR are crucial for the Cul3-mediated reduction of viral gene expression (Figure 5b). cDNA transfection efficiency was not affected by the depletion of Cul3 (Figure S3). Of note, the mutations in the HIV-1 NL4-3 ∆NF-κB mutant simultaneously abrogate the binding sites for the NFAT transcription factor. We therefore cannot definitely conclude that the Cul3 effect is dependent on the NF-κB sites alone. Overexpression of Cul3 in HEK293T cells, however, did not alter NFAT signaling, indicating that NFAT signaling is not involved in Cul3-mediated inhibition of viral gene transcription (Figure S4).

## 4. Discussion

The viral life cycle of HIV-1 is influenced by host proteins that either support or limit viral replication [2,5,6,7,8]. In the present study, we investigate the role of the E3 ubiquitin-protein ligase Cul3 in the context of HIV-1 infection. Unlike closely related protein family members Cul1, Cul4 and Cul5, which are hijacked by HIV-1 to support viral infection [22], our data demonstrate that Cul3 acts as a negative regulator of HIV-1 infection and further elucidate the mode of action by which Cul3 exerts this restrictive effect on HIV-1. Importantly, we show that (1) Cul3-silencing enhances, whereas Cul3-overexpression reduces HIV-1 infection in immortalized and primary cells, (2) the restrictive effect of Cul3 on infection can be assigned to a reduction of viral mRNA expression and (3) the presence of NF-κB/NFAT binding sites within the viral LTR, but not other transcription factor binding sites, are crucial determinants of this effect.

We found that Cul3 protein is highly expressed in primary and immortalized CD4+ T cells and monocyte-derived macrophages (MDMs), which represent relevant HIV-1 target cells. Depending on the specific antibody used, one or multiple bands were detected. The different bands potentially represent forms of Cul3 with different post-translational modifications, such as neddylation [51,52], that manifest as species with different molecular weights and hence an altered migration profile in the SDS-PAGE. Interestingly, overexpression of Cul3 in HEK293T cells reduced HIV-1 infection. The slightly more moderate inhibitory effect of overexpressed Cul3 on HIV-1 infection, when compared to a depletion of Cul3 via siRNA, could be explained by the fact that endogenous protein levels of Cul3 in HEK293T cells are relatively high, which sets a high base level and hence restricts the dynamic range of an overexpression approach. Additionally, while about 70% of the HEK293T cells got transfected with Cul3 cDNA (based on flow-cytometric analysis, data not shown), more than 90% of the cells were infected with HIV-1. This also potentially dampens the overall effect of Cul3 on infection in this experimental setup, as a significant number of cells are efficiently infected without Cul3 being overexpressed.

Based on data demonstrating that depletion of Cul3 resulted in an increase viral mRNA levels, we hypothesized that Cul3 might directly or indirectly be involved in the transcription of viral mRNA. HIV-1 relies on various human transcription factors that are able to bind at specific sites inside the viral LTR and thus support efficient mRNA transcription [44]. The pro-inflammatory NF-κB represents a well-characterized transcription factor, which HIV-1 relies on for synthesis of viral mRNA [30,32]. In order to complete its replication cycle in activated T cells, HIV-1 requires active NF-κB signaling to trigger efficient gene expression [53,54]. Furthermore, induction of the signaling pathway can also result in reactivation of virus from a latent state [55]. Our data indicate that the inhibitory effect of Cul3 on HIV-1 infection relies on the region in the viral LTR that harbors the NF-κB binding sites. Given previous reports indicating that the Cul3/Kelch-like ECH-associated protein 1 (KEAP1) E3 ubiquitin ligase complex targets IKKβ for ubiquitination and subsequent degradation, thereby preventing NF-κB translocation to the nucleus [28,56], we speculate that Cul3 limits viral mRNA expression by modulating NF-κB activation. Alternatively, due to the role of Cul3 in numerous cellular processes, it is possible that other targets of the E3 ubiquitin ligase complex affect viral replication. For example, Cul3 is known to target cyclin E for ubiquitination and subsequent degradation, thereby regulating G1 to S phase transition [26]. However, our data indicates that Cul3 exerts its effect on HIV-1 replication without affecting cell cycle progression (Figure 4d). Nevertheless, given the diverse roles of Cul3, we cannot exclude that this factor impacts HIV-1 replication through other known targets, such as 4E-BP1 [57] or EB1 [58], or unknown targets that regulate NF-κB or NFAT. Additional studies investigating potential targets of the regulatory activity of Cul3 are expected to provide important insight on how this factor limits viral replication. Moreover, further studies should provide a quantification of the impact of Cul3 on HIV-1 replication in primary CD4+ T cells.

Furthermore, our data indicates that, unlike HIV-1 restriction factors APOBEC3, tetherin and the SERINC proteins, which are degraded by HIV-1 encoded accessory proteins such as Vif, Vpu and Nef, respectively [59], Cul3 expression is not altered in the context of HIV-1 replication, indicating that HIV-1 does not antagonize the antiviral activity of Cul3 through protein degradation. Considering the role of Cul3 as a regulator of NF-κB signaling, it is important to note that the transcription factor NF-κB plays a dual role in HIV-1 replication, balancing both proviral and antiviral effects by not only acting as initiator of viral transcription, but also functioning as master regulator of the innate immune system [60,61,62,63,64]. The activation of NF-κB signaling leads to increased initiation of HIV-1 gene expression and thereby promotes viral replication. Conversely, this transcription factor is a key regulator of the type I IFN-mediated antiviral response [62]. Thus, the careful regulation and limitation of NF-κB activity is critical to maintain efficient viral replication in the absence of an excessive activation of innate immune responses. Therefore, Cul3 may exert a moderating effect on NF-κB activation, and thereby HIV-1 replication, that enables the virus to balance the requirement for NF-κB mediated transcription and detrimental antiviral responses. In addition, limiting the maximum rate of viral replication may even be advantageous to the virus, by promoting optimal virion production and release before the impact of cytopathic effects in infected cells starts to build up. This may suggest a hypothesis that Cul3-mediated suppression of HIV-1 replication is advantageous for viral pathogenesis, thus obviating a need for a viral countermeasure to this cellular restrictive mechanism.

In addition to its role in initiating efficient proviral transcription and mounting innate immune activation [62], NF-κB plays a key role in the regulation of HIV-1 latency. If NF-κB is inactive in an infected cell, the virus remains in a dormant state, which represents a major barrier to the eradication of the virus in infected individuals [65]. Thus, activating NF-κB to purge the virus from latent reservoirs, and then pharmacologically targeting the virus-expressing cells for eradication, represents a promising strategy to cure HIV/AIDS, termed “Shock and Kill” [66,67]. Targeting Cul3 using small-molecules may represent a novel strategy to boost viral gene expression of latent HIV-1 and introduce components of the ubiquitination pathway as a new class of pharmacological targets of latency reversal agents (LRAs).

Taken together, we show that the E3 ubiquitin-protein ligase Cul3 regulates NF-κB activation, and thereby affects LTR-mediated HIV-1 gene expression in infected target cells. Thus, our results introduce Cul3 as an important regulator of HIV-1 replication. Understanding how Cul3 restricts HIV-1 transcription could potentially lead the way towards new approaches for HIV/AIDS therapies and may provide novel strategies to clear the HIV-1 reservoir from infected individuals.

## Supplementary Materials

The following are available online at https://www.mdpi.com/2073-4409/9/9/2010/s1, Figure S1: Endogenously Cul3 is not degraded by HIV-1 in transfected HEK293T cells, Figure S2: Cul3 overexpression reduces NF-κB activity in transfected HEK293T cells, Figure S3: Cul3 depletion does not affect transfection efficiency; Figure S4: Cul3 overexpression does not reduce NFAT activity in transfected HEK293T cells.
