# Supplementary material for: The E3 Ubiquitin-Protein Ligase Cullin 3 Regulates HIV-1 Transcription
Source: Cells. 2020 Sep 1;9(9):2010. doi: 10.3390/cells9092010 (PMC7564853; doi:10.3390/cells9092010)
Supplement: Supplementary file 1 [file cells-09-02010-s001.pdf]

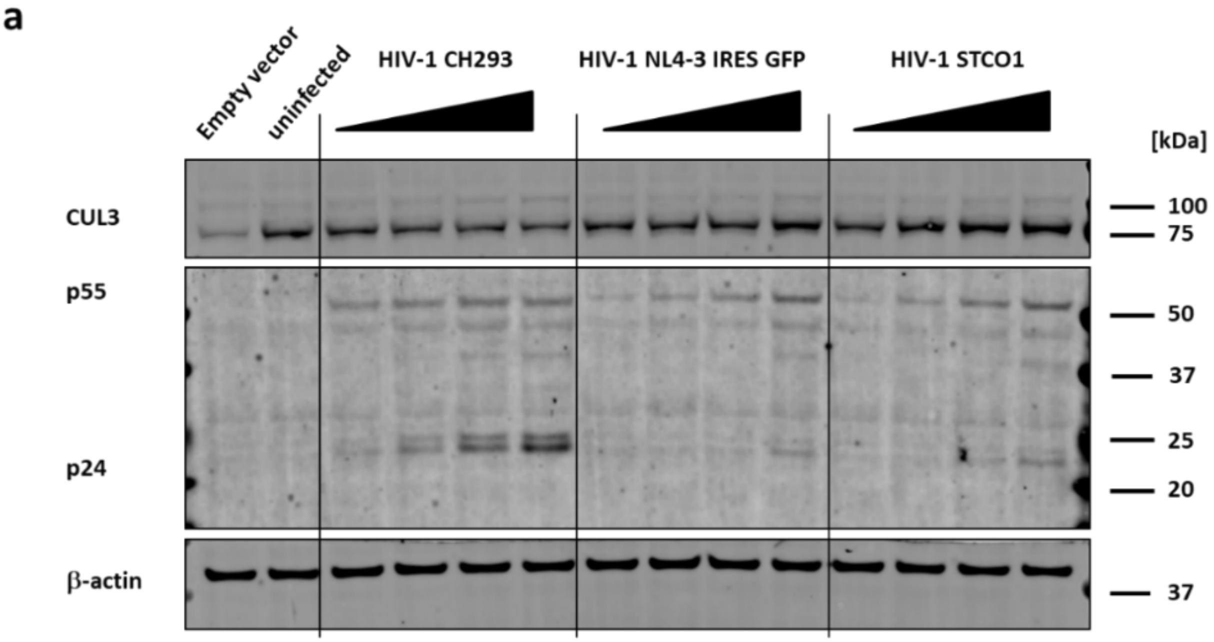
a
Empty vector
uninfected
HIV-1 CH293
HIV-1 NL4-3 IRES GFP
HIV-1 STCO1
[kDa]
CUL3
100
75
p55
50
37
25
p24
20
β-actin
37

b

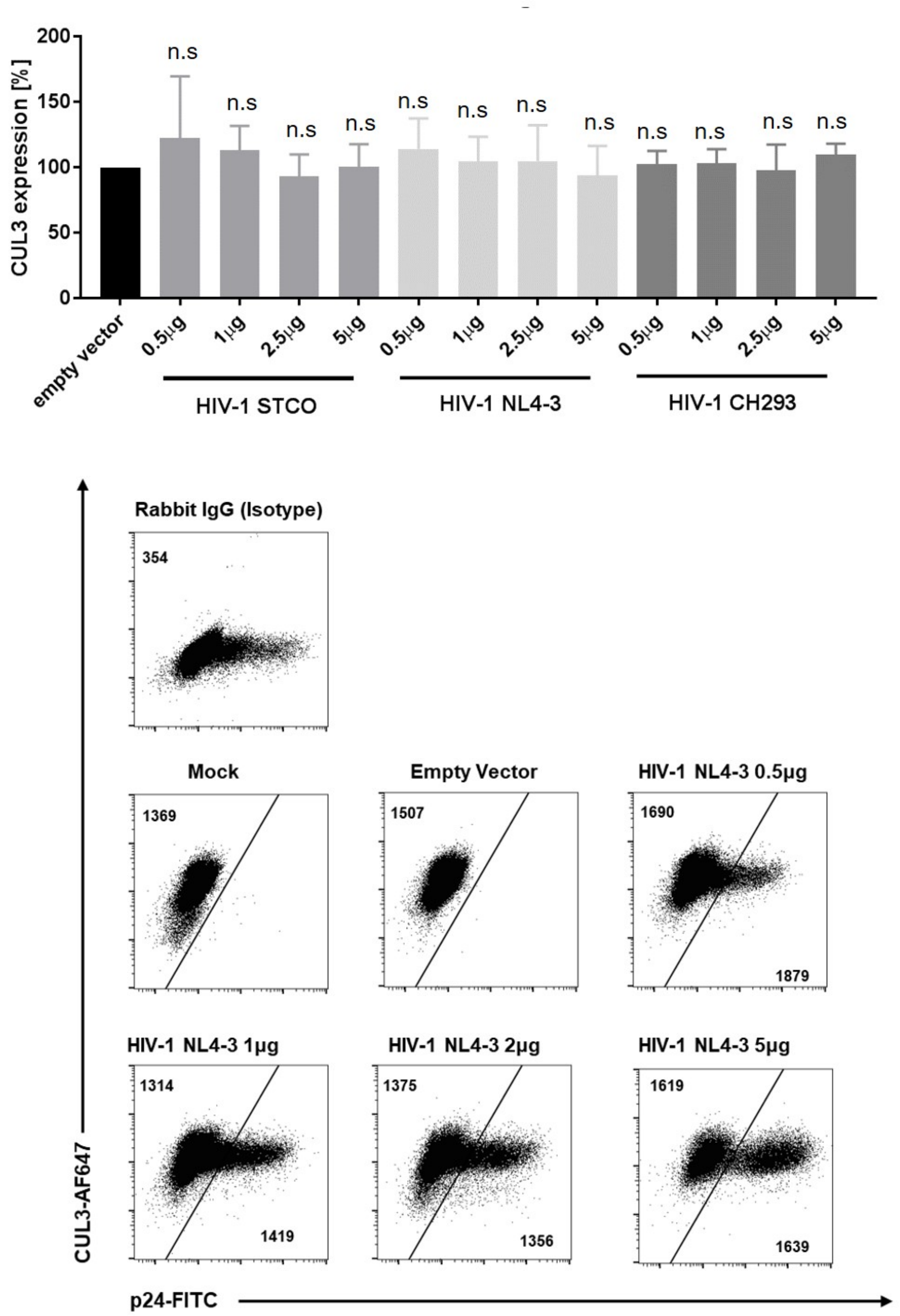
CUL3 expression [%]
200
150
100
50
0
n.s
empty vector
0.5µg
1µg
2.5µg
5µg
HIV-1 STCO
HIV-1 NL4-3
HIV-1 CH293
Rabbit IgG (Isotype)
354
Mock
1369
Empty Vector
1507
HIV-1 NL4-3 0.5µg
1690
1879
HIV-1 NL4-3 1µg
1314
1419
HIV-1 NL4-3 2µg
1375
1356
HIV-1 NL4-3 5µg
1619
1639
CUL3-AF647
p24-FITC

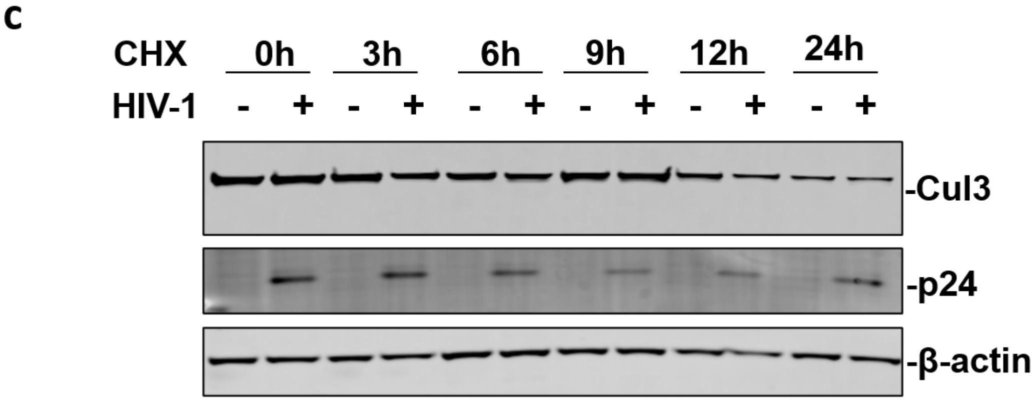

Figure S1. Endogenously Cul3 is not degraded by HIV-1 in transfected HEK293T cells. HEK293T cells were transfected with increasing amouts of the indicated proviral DNA (0.5 μg, 1.0 μg, 2.0 μg, 5.0 μg). (a) 40 h post transfection, cells were harvested, and Cul3 protein expression was analyzed by western blot using the indicated antibodies (n=3). (b) 40 h post transfection, Cul3 protein expression was monitored by two-color flow cytometry (upper panel) (n=2-5 +/-SD). Dot plots indicating the gating strategy and are shown in the lower panel. Numbers indicate mean fluorescence intensity (MFI). Isotype control consists of pooled cells from all samples. (c) HEK293T cells were transfected with HIV-1 NL4-3 proviral DNA (5.0 μg). 24 h post transfection, the cells were treated with cycloheximide (CHX; 100 μg/ml), and then collected at the indicated time point post-treatment. The expression of Cul3, HIV-1 p24 and β-actin was analyzed by western blot using the indicated antibodies.

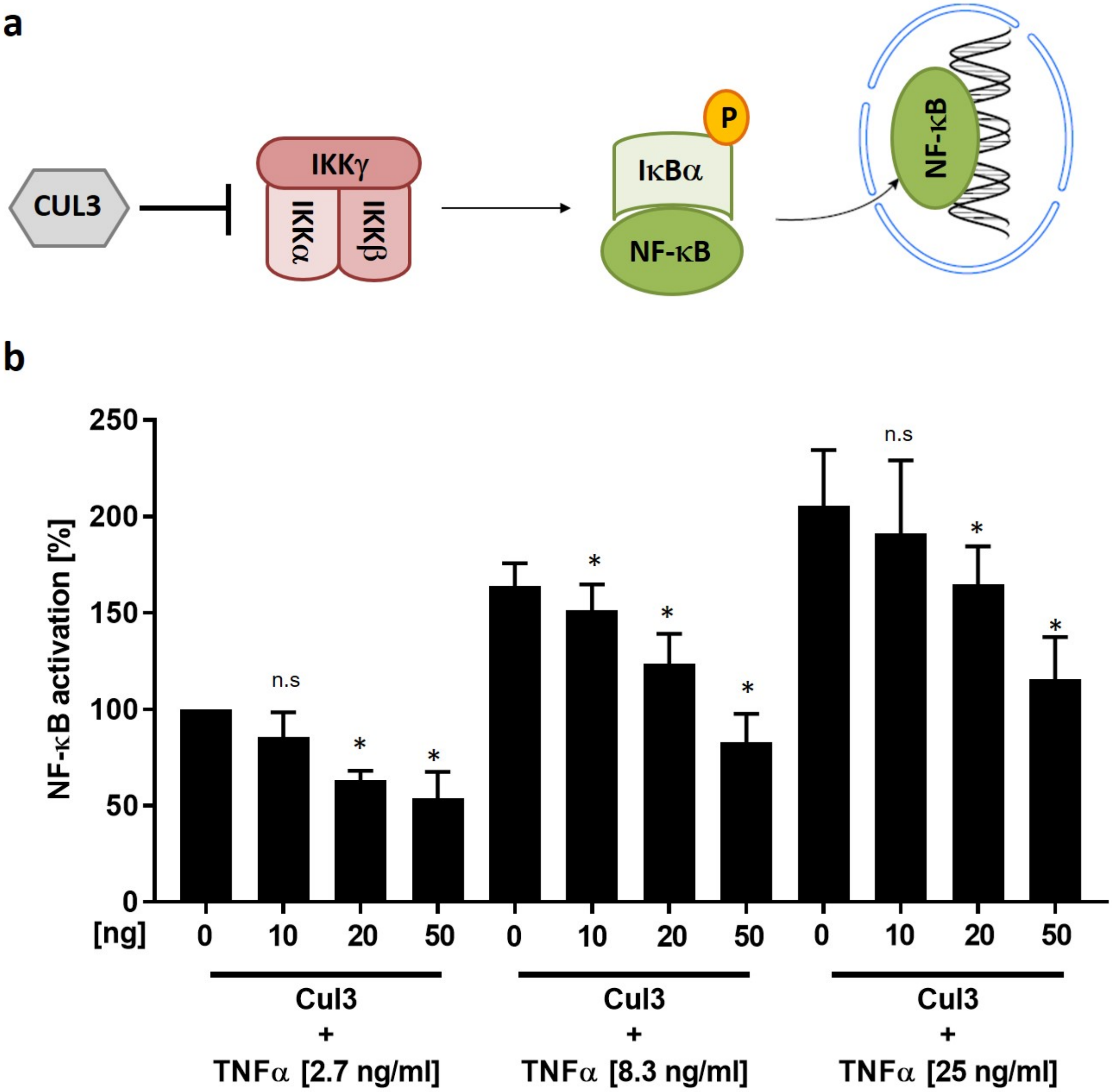

Figure S2. Cul3 overexpression reduces NF-κB activity in transfected HEK293T cells. (a) Cul3 negatively regulates NF-κB activation. (b) HEK293T cells were co-transfected with the indicated amounts of Cul3, 20 ng of an NF-κB-responsive firefly luciferase reporter construct and 5 ng of a Renilla luciferase reporter plasmid. 16 h post transfection, cells were treated with the indicated amounts of human TNFα. 24 h post stimulation, firefly luciferase activity was determined and normalized to the activity of the Renilla luciferase control plasmid. Data is shown as normalized to 0 ng Cul3 and 2.7 ng/mL TNFα (n=2-3 +/- SD).

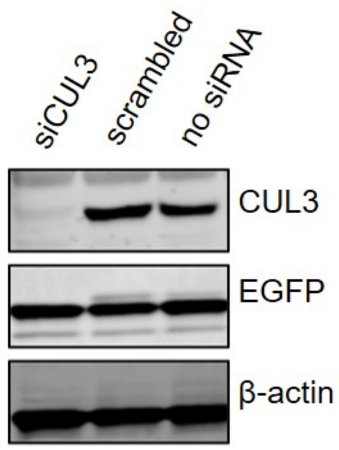

Figure S3. Cul3 depletion does not affect transfection efficiency. HEK293T cells, treated with an siRNA targeting CUL3 (siCUL3), a non-targeting siRNA (scrambled), or untreated (no siRNA) for 48 h, were transfected with the GFP-encoding pEGFP-C1. 24 hours post-transfection, the expression of GFP, CUL3, and β-actin was analyzed by western blot.

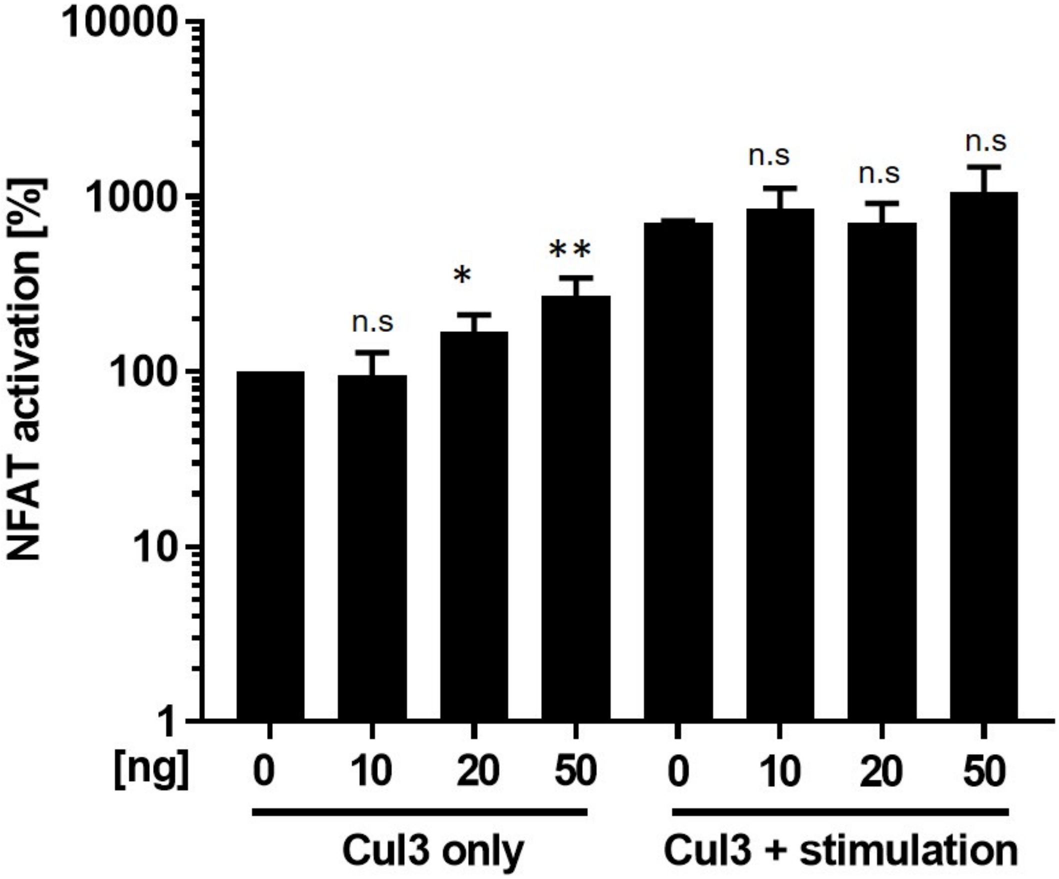

Figure S4. Cul3 overexpression does not reduce NFAT activity in transfected HEK293T cells. HEK293T cells were co-transfected with the indicated amounts of Cul3, 50 ng of an NFAT-responsive firefly luciferase reporter construct and 5 ng of a Renilla luciferase reporter plasmid. 24 h post transfection, cells were treated with human PMA (600 ng/mL) and Ionomycin (1 mM) ("Cul3 + stimulation") or kept untreated ("Cul3 only"). 17 h post stimulation, firefly luciferase activity was determined and normalized to the activity of the Renilla luciferase control plasmid. Data is shown as normalized to 0 ng Cul3, no treatment (n=3 +/- SD).
